# Engineering nanoparticles to silence bacterial communication

**DOI:** 10.3389/fmicb.2015.00189

**Published:** 2015-03-10

**Authors:** Kristen P. Miller, Lei Wang, Yung-Pin Chen, Perry J. Pellechia, Brian C. Benicewicz, Alan W. Decho

**Affiliations:** ^1^Microbial Interactions Laboratory, Department of Environmental Health Sciences, Public Health Research Center, Arnold School of Public Health, University of South CarolinaColumbia, SC, USA; ^2^Department of Chemistry and Biochemistry, JM Palms Center for Graduate Student Research, University of South CarolinaColumbia, SC, USA

**Keywords:** nanomedicine, nanoparticles, quorum sensing, quorum quenching

## Abstract

The alarming spread of bacterial resistance to traditional antibiotics has warranted the study of alternative antimicrobial agents. Quorum sensing (QS) is a chemical cell-to-cell communication mechanism utilized by bacteria to coordinate group behaviors and establish infections. QS is integral to bacterial survival, and therefore provides a unique target for antimicrobial therapy. In this study, silicon dioxide nanoparticles (Si-NP) were engineered to target the signaling molecules [i.e., acylhomoserine lactones (HSLs)] used for QS in order to halt bacterial communication. Specifically, when Si-NP were surface functionalized with β-cyclodextrin (β-CD), then added to cultures of bacteria (*Vibrio fischeri*), whose luminous output depends upon HSL-mediated QS, the cell-to-cell communication was dramatically reduced. Reductions in luminescence were further verified by quantitative polymerase chain reaction (qPCR) analyses of luminescence genes. Binding of HSLs to Si-NPs was examined using nuclear magnetic resonance (NMR) spectroscopy. The results indicated that by delivering high concentrations of engineered NPs with associated quenching compounds, the chemical signals were removed from the immediate bacterial environment. In actively-metabolizing cultures, this treatment blocked the ability of bacteria to communicate and regulate QS, effectively silencing and isolating the cells. Si-NPs provide a scaffold and critical stepping-stone for more pointed developments in antimicrobial therapy, especially with regard to QS—a target that will reduce resistance pressures imposed by traditional antibiotics.

## Introduction

Excessive use of antibiotics has resulted in widespread bacterial resistance and poses a significant public health threat. Non-cytotoxic methods for controlling infections, such as interference in bacterial communication, are necessary to fight pathogenic infections while limiting the risk of antibiotic resistance (Rasko and Sperandio, 2010; Schuster et al., 2013). Quorum sensing (QS) is a chemical communication scheme used by bacteria to coordinate their activities within a biofilm, and it regulates many translational features that make bacteria dangerous (Davies, 1998; Ng and Bassler, 2009; Hoiby et al., 2010). One of the most significant challenges in the treatment of chronic infections is the effective delivery of drugs to bacterial biofilms. The importance of nanoparticle-based therapies is imperative to the treatment of biofilm infections. Biofilms are surface-associated bacterial communities living in a highly-organized structure at a liquid interface (Costerton et al., 1987), and have been estimated to be responsible for 80% of hospital-acquired infections (Davies, 2003). The protective nature of bacterial biofilms makes them able to physically limit antibiotic penetration (Stewart et al., 2001; Canton and Morosini, 2011), quickly regulate multidrug efflux pumps and stress response genes (Davies, 2003; Hoiby et al., 2010), induce a biofilm-specific phenotype (Allison et al., 2011), and easily trade and enhance antibiotic resistance genes among their bacterial members (Madsen et al., 2012).

**Figure 1:**
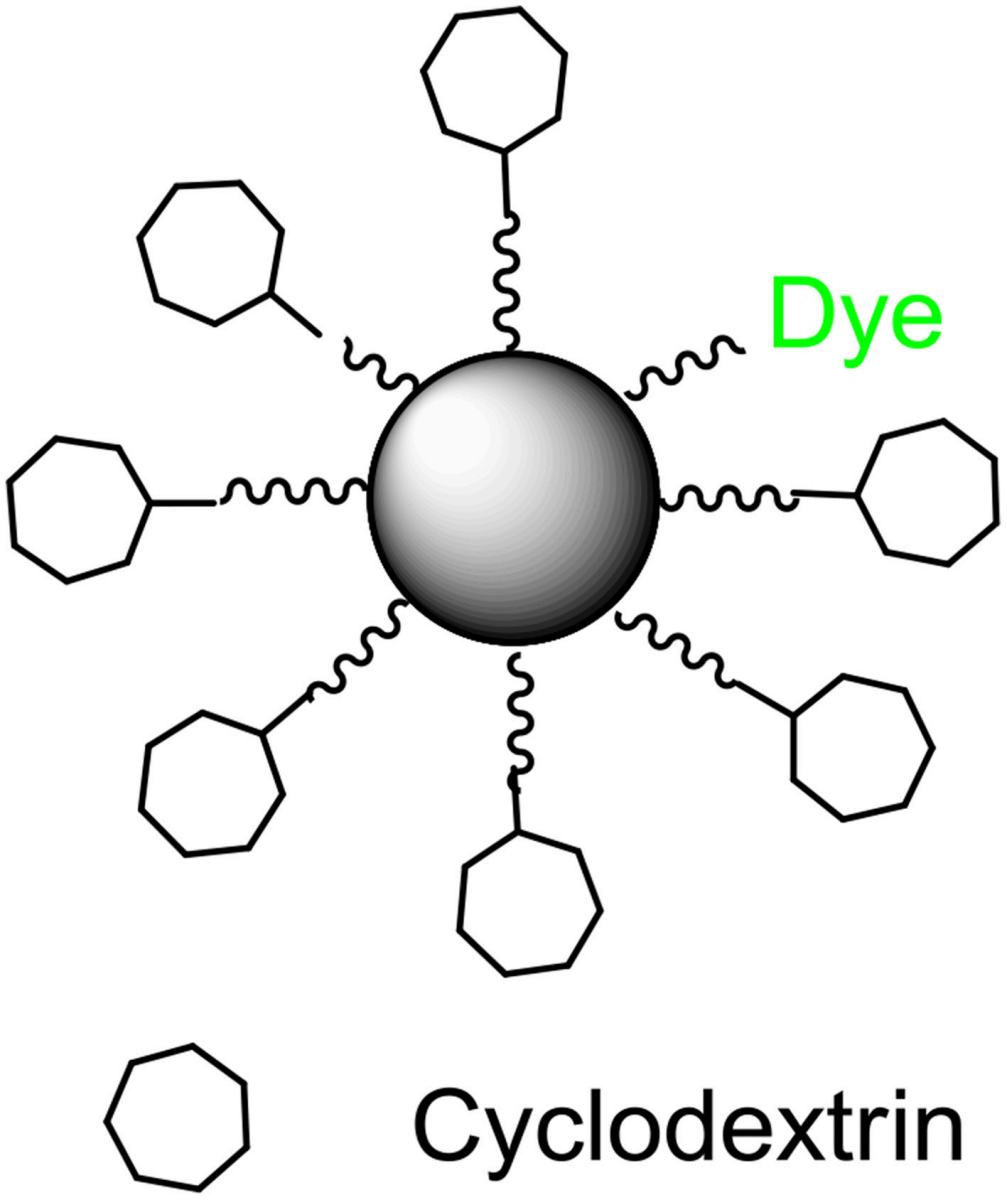
**Schematic of β-cyclodextrin coated silicon dioxide nanoparticle**.

**Figure 2:**
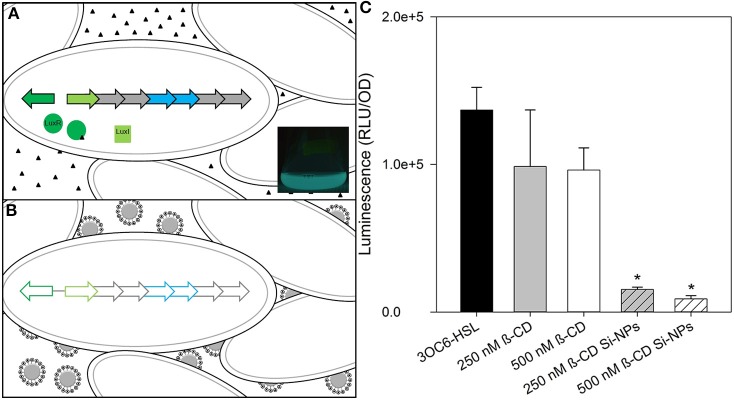
**Schematic of nanoparticle-based silencing of bacterial quorum sensing (QS). (A)** Diagram of the Gram-negative bacterium *Vibrio fischeri lux* operon used in luminescence during QS using acyl-homoserine lactones (HSL). Triangles represent 3OC6-HSL. LuxR/3OC6-HSL complex initiates bilateral transcription of *lux* operon. LuxI produces 3OC6-HSL. Inset: A one-liter *V. fischeri* culture flask luminescing after treatment with HSLs. **(B)** Diagram of *V. fischeri* during nanoparticle treatment. Binding of HSLs by Si-NPs quenches QS and subsequent gene expression in *lux*. **(C)** Changes in maximum bioluminescence by *V. fischeri* during exposure to 2 μ M 3OC6-HSL, with either β-CD or β-CD functionalized Si-NPs. Error bars represent standard error of the mean (*n* = 3). Asterisks indicate significance (*P* = 0.05) by ANOVA.

Silicon dioxide nanoparticles (Si-NPs) have a broad range of applications within the biomedical and industrial fields due to their high biocompatibility and controllable particle size (De et al., 2008; Malvindi et al., 2012; Wang and Benicewicz, 2013). Inspired by developments in the field of drug delivery, we have designed Si-NPs (Figure 1) to carry compounds that will quench signal molecules. Removal of signals from the immediate bacterial environment prevents the molecule from reaching its cognate receptor, thus inhibiting the signal/receptor interaction, and interfering with down-stream regulation. We hypothesized that this process will quench signal molecules, “turn off” QS, and silence bacterial communication (schematic seen in Figures 2A,B).

β-cyclodextrin (β-CD) has been shown to non-specifically bind *N*-acyl-L-homoserine lactone (HSL) molecules (Kato et al., 2006), a common class of signal molecules produced by Gram-negative bacteria. β-CD consists of seven glucopyranose units bound by α-1,4-glycosidic linkages. This molecule takes the shape of a truncated cone with a hydrophilic exterior and a slightly hydrophobic interior. β-CD was chosen as a model compound because the formation of a CD complex can be detected and because the reactivity of a guest molecule in a CD complex is subsequently modified (Szejtli, 1998). In many cases, the CD accelerates the various reactions associated with the guest molecule and modifies the reaction pathway. Herein we present a new approach to disrupting bacterial QS with Si-NPs that have been surface-functionalized with β-CDs.

To test the quorum quenching function of the Si-NPs, the marine bacterium *Vibrio fischeri* was employed as a model. *V. fischeri* relies on *N*-acyl-L-homoserine lactone molecules to trigger coordinated activities such as colonization and bioluminescence in a threshold dependent manner during QS (Kaplan and Greenberg, 1985). Specifically, *V. fischeri* synthesizes and responds to *N*-3-oxo-hexanoyl-L-homoserine lactone (3OC6-HSL) (Eberhard et al., 1981; Engebrecht and Silverman, 1984) and *N*-octanoyl-L-homoserine lactone (C8-HSL) (Engebrecht et al., 1983) via the *lux* operon, which is also responsible for the proteins that synthesize luminescent luciferase (Stevens et al., 1994). HSLs are also used by pathogenic bacteria such as *Actinobacillus*, *Salmonella*, *Pseudomonas*, and other *Vibrio* species to regulate their respective QS genes (Defoirdt, 2013; LaSarre and Federle, 2013; Li et al., 2013b).

In this study, the fold change in the *luxA* and *luxR* transcription of *V. fischeri* was quantified to determine the activity of the *lux* operon during exposure to β-CD. LuxA forms the alpha subunit of luciferase and was used to monitor bacterial luminescence. LuxR is a receptor for both 3OC6-HSL and C8-HSL, and initiates the *lux* operon, and was used to monitor signal production. In the natural ocean habitat, this QS function allows *V. fischeri* to establish a symbiotic relationship with the Hawaiian bobtail squid (*Euprymna scolopes*) and provide counterillumination for the squid (Visick et al., 2000). Environmental conditions are essential for the proper functioning of *V. fischeri* bioluminescence. A symbiotic culture produces approximately 1000-fold brighter bioluminescence and more 3OC6-HSL than cultured cells at the same density (Boettcher and Ruby, 1990). Therefore, HSLs must be added to the cultures to induce QS and visible bioluminescence *in vitro*.

In this study we have engineered nanoparticles that weaken the communication network of bacteria, rather than directly target the viability of cells. The functionalized Si-NPs bind small signal molecules as they diffuse between cells, and subsequently block bacterial QS. Using growth and transcription studies, we demonstrate that this novel technology can be used to directly influence bacterial communication and activities regulated by QS, and offer a promising tool that can be further developed to unravel the virulence of pathogenic bacteria. *V. fischeri* was used in its planktonic form to establish the utility of the functionalized NPs in a simple environment. Also, this bacterium was used because its QS mechanism is well-established in the literature and can be readily detected (by luminescence) in the laboratory. A biofilm model has not yet been employed here because of the inherent complexity found in a biofilm, and the subsequent difficulty in monitoring the QS response.

## Results

### Si-NPs carry β-cyclodextrin

The unique properties of surface-functionalized Si-NPs make them ideal for targeting the multiple avenues of bacterial defenses and weakening an infection. Surface functionalization plays a critical role in tailoring the properties of Si-NPs via well-developed surface chemistry (Li et al., 2013a, 2014). For this study, the synthesized β-CD functionalized Si-NPs were purified via dialysis to remove un-reacted (i.e., free) β-CD molecules, as confirmed by ^1^H NMR (Supplementary Figure 1). Thermogravimetric analysis (TGA) confirmed that the monolayer of β-CD accounted for 2.78% of the total weight of particles and indicated a surface graft density of 0.27 and 0.11 groups/nm^2^ for the 15 and 50 nm nanoparticles, respectively (Supplementary Figure 2).

### β-cyclodextrin binds HSLs in solution

NMR spectroscopy was used to determine binding strengths between β-CD and HSLs. NMR diffusion experiments demonstrated that both C8-HSL (produced by *V. fischeri*) and C6-HSL (produced by *Pseudomonas aeruginosa*) could bind to β-CD in a 1:1 ratio; however, there was no evidence that 3OC6-HSL (also produced by *V. fischeri*) formed a complex with β-CD. Further calculations revealed the binding and dissociation constants of the experimental complexes. The binding between the HSLs and β-CD accounted for only 17% of C6-HSL and 35% of C8-HSL at any one time in the 1:1 solution (Supplementary Tables 1, 2). Despite this relatively low binding, we proceeded to quantify the quenching ability of β-CD *in vitro*. Using growth and luminescence studies we observed the influence of β-CD on *V. fischeri* bioluminescence.

### Nanoparticles enhance ability of β-cyclodextrin to quench HSLs and dim bioluminescence

*V. fischeri* cultures grown in the presence of 2 μ M 3OC6-HSL exhibited strong bioluminescence and normal growth. When treated with concentrations of free β-CD ranging from 250 nM to 7 mM, the growth study showed that exposure to 2 mM β-CD resulted in the most significant decrease in bioluminescence (Supplementary Figure 3). A preliminary analyses of β-CD functionalized Si-NPs with 2 μ M 3OC6-HSL demonstrated that the β-CD moietie was significantly more-effective at dimming bioluminescence of *V. fischeri* when functionalized to a Si-NP than it was as a free-compound (Figure 2C). Statistical significance for all experiments was determined by a repeated measures analysis of variance test followed by an *aposteriori* pairwise muliple comparision procedure (SigmaPlot, Systat Software, San Jose, CA). Because 2 μ M 3OC6-HSL likely exceeds the expected HSL production of symbiotic *V. fischeri* cultures [which is unknown, but luminescence is induced in the nanomolar range (Lupp and Ruby, 2005)], and does not induce the *lux* operon like C8-HSL, we altered the HSL conditions to more-closely mimic the natural environment.

Growth experiments were repeated with *V. fischeri* cultured with 125 nM 3OC6-HSL and 0.25 nM C8-HSL. Optical density and bioluminescence were monitored throughout growth to determine the impact of free and functionalized β-CD. All cultures were grown in defined minimal medium (Studer et al., 2008), and therefore were not in a rich nutrient environment. Additional growth studies indicated that the bacteria did not thrive on β-CD or the various Si-NPs. Also, when all carbon sources in the defined minimal medium were replaced with β-CD, no growth occurred. Cultures grown without the addition of HSLs produced negligible levels of bioluminescence. Therefore, β-CD, with or without NPs, did not influence the growth or relative health of the cells.

**Figure 3:**
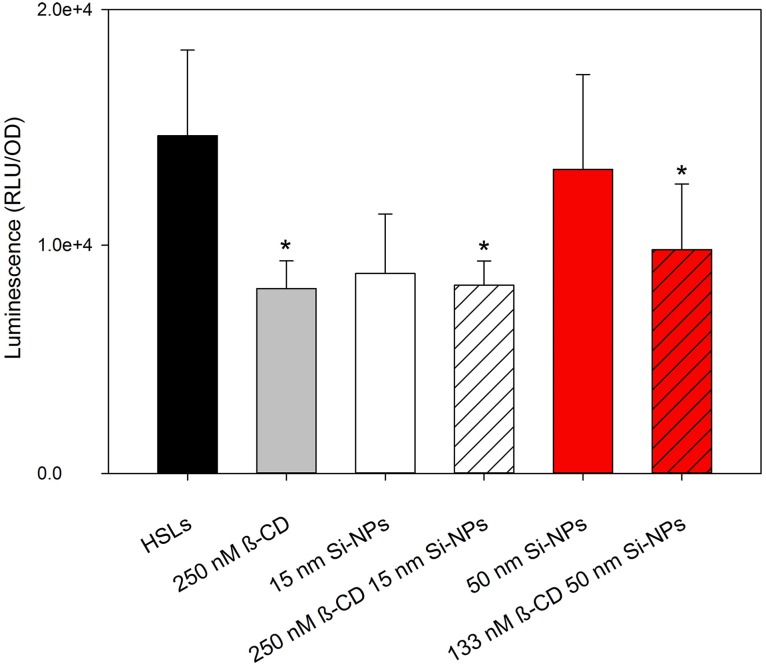
**Mean relative maximum bioluminescence per OD (600 nm) of *V. fischeri* during exposure to 125 nM 3OC6-HSL and 0.25 nM C8-HSL treated with 250 nM β-CD, bare 15 nm Si-NPs, 155 nM β-CD functionalized 15 nm Si-NPs, bare 50 nm Si-NPs, or 133 nM β-CD functionalized 50 nm Si-NPs**. Error bars represent standard error of the mean (*n* = 3). Asterisks indicate significance (*P* = 0.05).

*V. fischeri* cultures grown in the presence of 125 nM 3OC6-HSL and 0.25 nM C8-HSL produced measureable bioluminescence; although, bioluminescence in general was lower compared to the cultures treated with 2 μ M 3OC6-HSL (as indicated by the different scales in Figures 2C, 3). Our study found that at environmentally-relevant levels of HSLs, bioluminescence was significantly reduced *in vitro* by β-CD (*P* = 0.05). By functionalizing β-CD to 50 nm NPs, 133 nM β-CD was able to produce the same result as a 2x higher concentration of free 250 nM β-CD (Figure 3). We found that 15 nm Si-NPs with or without β-CD decreased bioluminescence to the same extent, and thus may not have a significant role in dimming luminescence.

The long half-life (relative to a 12-h growth experiment) of luciferase likely contributed to the uncertainty in discerning subtle changes in QS over time. To further examine the role of functionalized and free β-CD in *V. fischeri* QS, we then examined changes in transcription of the *lux* operon during exposure to β-CD and β-CD functionalized Si-NPs.

### *Lux* operon down-regulated in presence of β-CD functionalized Si-NPs

Bioluminescence in *V. fischeri* is generated by the *lux* operon and is activated by 3OC6-HSL and C8-HSL. Transcription of the *lux* operon results in the production of luciferase, the enzyme responsible for bioluminescence. Table 1 summarizes the fold-changes calculated for *luxA* and *luxR* from the C_t_ values generated by qPCR with the Livak method (ΔΔC_t_) of both HSL-treated cultures.

**Table 1:** **Calculated fold change of transcription by *V. fischeri* after treatment exposures to 125 nM 3OC6- and 0.25 nM C8-HSLs**.

	*luxR*	*luxA*
no treatment (control)	−1.4	4.8
250 nM β-CD	−1.7	1.7
2 mM β-CD	−97.8	−39.1
15 nm bare-NPs	−250.4	−432.9
15 nm 155 nM β-CD-NPs	−245.7	−629.77
50 nm bare-NPs	−1723.4	−365.8
50 nm 133 nM β-CD-NPs	−2125.5	−2171.1

Negative values indicate down-regulation.

The quantity of transcripts produced by untreated cultures (controls) and cultures treated with 250 nM β-CD were not markedly different, indicating that low concentrations of free β-CD are ineffective in down-regulating *luxA* and *luxR* at environmental levels of HSLs (data not shown). The addition of much higher (i.e., 2 mM) β-CD significantly reduced the expression of *luxA* and *luxR* transcripts, as indicated by the calculated fold changes (*P* = 0.05). In an attempt to more efficiently deliver β-CD to the bacterial cell, low concentrations of β-CD were applied via Si-NPs. As Table 1 indicates, both the functionalized and non-functionalized 15 nm Si-NPs reduced the expression of *luxA* and *luxR* transcripts to a similar extent. The data agree with the growth study, indicating that the influence of 15 nm Si-NPs functionalized with or without 155 nM β-CD is indistinguishable. Conversely, treatment with the larger nanoparticles (i.e., functionalized and non-functionalized 50 nm Si-NPs) resulted in significantly different fold changes (*P* = 0.05). The 133 nM β-CD, when bound to functionalized 50 nm Si-NPs, resulted in the greatest down-regulation of *luxA* and *luxR* transcripts out of all treatments, demonstrating the strong utility of this size of functionalized Si-NPs. The discontinuity of the bioluminescence measurements between the growth and transcription studies was likely influenced by the relatively long half-life of bioluminescence, which contributed to an inability to discern differences using bioluminescence during growth.

## Discussion

The bioluminescence and transcription studies indicate that the 15 nm and 50 nm Si-NPs resulted in different levels of QS, although both Si-NPs were synthesized using the same method. Based on the nanoparticle synthesis methods and TGA analyses, we determined that the 15 nm Si-NPs carry 0.06 μmol β-CD/mg Si-NP complex, while the 50 nm Si-NPs carry 0.15 μmol β-CD/mg Si-NP complex, indicating that the surface densities of the two Si-NPs are different. Additionally, the surface area of the 50 nm Si-NPs is more than ten times the surface area of the 15 nm Si-NPs, which may have provided for more space between individual β-CD molecules and allowed for more efficient binding with the HSLs. Given that a 50 nm Si-NP can carry 1.7 times more β-CD than a 15 nm Si-NP, a higher dose of 15 nm Si-NPs was used to treat the samples to achieve comparable levels of β-CD. Three major possibilities exist to explain the difference in results. First, the higher concentration of Si-NPs aggregated in solution, making more of the functional groups unavailable on the smaller Si-NP. Alternatively, the β-CD may have been too dilute among the 15 nm Si-NPs and therefore did not act differently than dissolved β-CD. Lastly, it is possible that the 15 nm Si-NPs behave differently than 50 nm Si-NPs in an aqueous solution (Vertegel et al., 2004; Jiang et al., 2008). It has been shown that the surface modifications of silica affect the dispersion of Si-NPs, and that smaller-sized Si-NPs have unique physicochemical properties and optical absorption characteristics (Rahman et al., 2009). Therefore, smaller nanoparticles with a low surface density may behave differently than larger nanoparticles with a higher surface density. In summary, it is likely that the β-CD dose carried by the 15 nm Si-NPs behaved differently than the 50 nm Si-NPs, and therefore was unable to impact QS more significantly than free β-CD. The larger 50 nm Si-NPs, in contrast, were able to carry a higher dose of β-CD on fewer nanoparticles, and directly influenced QS in *V. fischeri*.

Previous studies have reported the use of QS antagonists and inhibitors to successfully interfere with bacterial QS (Dong et al., 2007; Hong et al., 2012; Stacy et al., 2012; Welsh et al., 2015). QS antagonists are often plant- and algal-based compounds that bind to LuxR-type receptors and prevent the complex from initating QS. Alternatively, QS inhibitors include both natural and synthetic compounds that either degrade (i.e., enzymes) or inhibit HSLs. Many propose that these anti-virulence strategies will decrease selective pressure; however, the risk of antibiotic resistance cannot be eliminated given the rapid rate of bacterial revolution (Rasko and Sperandio, 2010). Our nanoparticle-based QS inhibitors also reduce the risk of antibiotic resistance by delivering high concentrations of the quenching compound without excessive loss of the compound into the microbial environment. This method allows us to specficially target harmful bacteria while reducing side effects often incurred by broad-spectrum antibiotics. By delivering a highly concentrated compound to a localized environment, the bacteria are treated quickly and have less time to respond and adapt. Our study represents one of the first attempts to deliver QS quenching compounds to bacteria via functionalized nanoparticles. Other studies have used antimicrobial nanoparticles, such as silver and titanium dioxide, to kill bacteria or eliminate an infection. However, bacteria have evolved multiple resistance mechanisms against these nanoparticles (Rizzello and Pompa, 2014). Our study is significant because it utilizes the size and capacity of nanoparticles and the strength and relative safety of a QS quenching compound to target and weaken bacteria. This technology could be potentially redesigned to carry highly specific compounds in an effort to further reduce selective pressure and eliminate harmful side effects.

In this study, we have demonstrated that functionalized silica nanoparticles can be used to enhance the role of quenching agents *in vitro*. Specifically, we demonstrated that β-CD is able to bind HSLs and down-regulate bacterial QS genes. We have found that the quenching ability of β-CD was much greater on functionalized 50 nm Si-NPs, and provides a strong model for future designs. Here, with the advent of nanotechnology, we have demonstrated a unique ability (and tool) to specifically target bacterial communication, which may be used to penetrate biofilms and eliminate infections.

## Materials and methods

### Diffusion study

NMR diffusion measurements were performed on a Varian Mercury/VX 400 spectrometer operating at 400.273 MHz (^1^H). Pulsed-field gradient spin echoes with varying gradient intensity were collected with the Doneshot (Pelta et al., 2002) pulse sequence that was included with VNMRJ 2.2D software. The standard Varian Performa I pulsed field gradient amplifier and 5 mm broadband probe were capable of producing a maximum of 20 gauss/cm field gradients. Spectra were taken at ambient temperature with bipolar gradient pulses of 4 ms and a diffusion delay of 100 ms. All data processing was done with the Varian DOSY software package.

### Bacteria and growth media

*V. fischeri* JB10 is a derivative of the ES114 strain that contains a chromosomal *gfp* reporter within the *lux* operon, resulting in *luxI-gfp-CDABEG* (Bose et al., 2007). The strain was prepared by and obtained from Professor Eric Stabb (Univ. Georgia, Athens, GA). *V. fischeri* JB10 was cultured from a glycerol stock and grown in marine broth (Difco 2216) to exponential phase. The cells were then transferred to defined minimal medium (Studer et al., 2008) for an optical density of approximately 0.1 (Abs_600nm_) for the growth and RNA extraction experiments.

### Bioluminescence response during exposure to nanoparticles

*V. fischeri* JB10 was grown in marine broth to exponential phase and then transferred (0.5%) to a defined minimal medium (Studer et al., 2008). For growth experiments, 96-well plates were incubated at 28°C in a Victor X Multilabel plate reader for 24 h. Optical density was measured at 600 nm and GFP fluorescence was measured with a 485/20 nm excitation filter and a 528/20 nm emission filter. Bioluminescence was also measured. All measurements were conducted every 2 h for 24 h following 10 s of vigorous shaking. The total volume of each well was 200 μ L, which included either 250 nM β-CD, 2 mM β-CD, 15 nm SiO_2_ nanoparticles (2.75 × 10^−3^ mg/mL NPs), 15 nm SiO_2_ nanoparticles functionalized with 155 nM β-CD (2.75 × 10^−3^ mg/mL NPs), 50 nm SiO_2_ nanoparticles (8.9 × 10^−4^ mg/mL NPs), or 50 nm SiO_2_ nanoparticles functionalized with 133 nM β-CD (8.9 × 10^−4^ mg/mL NPs). Cultures also included 125 nM 3OC6-HSL, 0.25 nM C8-HSL, or both 125 nM 3OC6-HSL and 0.25 nM C8-HSL. All treatments were added at time zero. A growth experiment was also performed with 600 nm SiO_2_ particles (data not shown, no cell growth occurred), α-cyclodextrin, 2-hydroxypropyl-β-CD, and methyl-β-CD. Cyclodextrins were obtained from Sigma Aldrich.

### Quantitative PCR and transcript analysis: lux transcription during exposure to nanoparticles

*V. fischeri* JB10 was grown in marine broth to exponential phase and then transferred (1%) to a defined minimal medium (Studer et al., 2008). Cultures were grown at 28°C and 200 rpm in 250 mL shake flasks with a working volume of 50 mL for 8 h. Treatments were added at 8 h and exposure/growth continued for 4 h. At 12 h of total growth, 2 mL of cells were harvested by centrifugation at 2000 × g (5 min). Cells were treated with 2 mg/mL lysozyme in TE buffer for 10 minutes and then homogenized with a needle and syringe. RNA was extracted with PureLink RNA Mini Kit (Ambion) and on-column DNA digestion was performed with PureLink PCR Micro Kit (Invitrogen). qPCR was performed on a BioRad CFX96 Real Time System with a C1000 Thermal Cycler. Data was analyzed by the Livak method (ΔΔC_t_) and fold changes were determined by using 2^−ΔΔCt^.

Primers were designed for *luxR* and *luxA* with NCBI's Primer-BLAST. Primer sequences for *luxA* were ATCCCCATCTTCGTGAACGG and ACAGAACATGGCCACGACAT. Primer sequences for *luxR* were CGTGGGCGAGTGAAGGAAAA and TGGCGCCAGTTAAAATTGCT. Primer sequences for the *16S rRNA* gene were GTTTGATCATGGCTCAGATTG and CTACCTTGTTACGACTTCACC (Hoffmann et al., 2010). The *16S rRNA* gene was used as the reference gene. All primers were ordered from Integrated DNA Technologies (Coralville, Iowa). Genomic DNA from *V. fischeri* JB10 was amplified with the primers and sequenced by Selah Genomics (Columbia, SC) to check the accuracy of the primers.

### Reagents for nanoparticle synthesis

All chemicals were obtained from Fisher or Acros and used as received unless otherwise specified. 3-aminopropyldimethyl-ethoxysilane was obtained from Gelest, Inc. (Morrisville, PA).

### Nanoparticle characterization

The nanoparticles were characterized according to methods establish by Wang and Benicewicz (2013) and Wang et al. (2014). Infrared spectra were determined with a BioRad Excalibur FTS3000 spectrometer. UV-vis spectra were measured with a Perkin-Elmer Lambda 4C UV-vis spectrophotometer. Infrared spectra were recorded with a Perkin-Elmer Spectrum 100 spectrometer. TGA was conducted using a SDT Q600 TGA system (TA Instruments) with a temperature ramping from 25 to 900°C at a rate of 10°C/min under nitrogen.

### Preparation of monolayer dye-labeled β-CD functionalized silica nanoparticles

A method has recently been developed for controlling the density of surface carboxylic acid functional groups coated onto the synthesized silicon dioxide Si-NPs (Cash et al., 2012; Wang and Benicewicz, 2013). Using this method, we coated a monolayer of β-CD onto the fluorescent Si-NPs via a coupling reaction between the β-CD hydroxyl groups and the carboxylic acids on the monolayer of dye-labeled Si-NPs. The carboxylic acid coated Si-NPs were prepared based on a ring-opening reaction between succinic anhydride and amino-functionalized silica Si-NPs with surface graft densities ranging from 0.10 to 0.65 groups/nm^2^. Thus, the graft density of β-CD functionalized Si-NPs were tailored by varying the feed ratio between bare Si-NPs and amino-silane compound. Specifically, a dimethylformamide (DMF) solution of β-CD (70.56 mg, 62.16 μmol), N,N′-Dicyclohexylcarbodiimide (DCC, 10.3 mg, 49.73 μmol) and 4-Dimethylaminopyridine (DMAP, 0.5063 mg, 4.144 μmol) were added to a 15 mL DMF solution of dye-labeled carboxylic acid-functionalized silica nanoparticles (graft density: 0.24 groups/nm^2^, 0.7281 g). The reaction was stirred at room temperature overnight. The reaction solution was then poured into 200 mL ethyl ether followed by centrifugation at 3000 rpm for 5 min. The recovered particles were then redispersed in 20 mL of ethanol and subjected to dialysis process to further remove impurities. The dye-labeled β-CD coated silica nanoparticles were finally dissolved in ethanol/water mixture solvents for further use. The preparation of dye-labeled monolayer carboxylic acid functionalized silica nanoparticles was based on our previous work (Cash et al., 2012).

## Statistical analysis

Statistical significance for all experiments was determined by a repeated measures analysis of variance test followed by an *aposteriori* pairwise muliple comparision procedure (SigmaPlot, Systat Software, San Jose, CA).

### Conflict of interest statement

The authors declare that the research was conducted in the absence of any commercial or financial relationships that could be construed as a potential conflict of interest.
