# Supplementary material for: Engineering nanoparticles to silence bacterial communication
Source: Front Microbiol. 2015 Mar 10;6:189. doi: 10.3389/fmicb.2015.00189 (PMC4354405; doi:10.3389/fmicb.2015.00189)
Supplement: Supplementary file 1 [file DataSheet1.DOCX]

**Supporting Information**

Engineering Nanoparticles to Silence Bacterial Communication

*Kristen P. Miller, Lei Wang, Yung Pin Chen, Perry J. Pellechia, Brian C. Benicewicz, & Alan W. Decho^*^*

**NMR detects binding of N-acyl homoserine lactone and β-cyclodextrin**

The NMR diffusion measurements were used to provide insight into the binding of *N*-octanoyl-*L*-homoserine lactone (C8-HSL) to both α-cyclodextrin and β-cyclodextrin. The data for various concentrations of C8-HSL and CD are summarized in Supplementary Table 2. The observed diffusion coefficients (*D*) are the average values that were determined for each ^1^H resonance arising from the two compounds. While the *D* of the cyclodextrins in each solution remains unchanged (within experimental error), the *D* of C8-HSL decreases towards the *D* of CD as the ratio of CD to HSL is increased. In the case of a large excess of CD, the equilibrium of C8-HSL shifts to the bound form and the *D* of C8-HSL is the same as the CD. This indicates that all of the C8-HSL is in the bound form.

It is important to note that NMR diffusion data for the HSL can be fit with a single exponential function and there is no evidence of two diffusing forms, i.e., free and bound. Therefore, the data suggests that during the timeframe of the NMR experiments (100s msec) the HSL is rapidly moving between free solution and bound into the CD and an equilibrium of the two forms exists.

The observed diffusion coefficient (*D_o_*) for the C8-HSL in these mixtures is a weighted average between the free diffusion coefficient (*D_f_*) and that of the bound from (*D_b_*) ^(Derrick et al., 2002)^

*D_o_ = F_f_D_f_ + F_b_D_b_* (1)

where *F_f_* is the fraction of C8-HSL that is free and *F_b_* is the fraction that is bound. The dissociation constant can be calculated from the measured diffusion coefficient and the starting concentrations of the two species by:

| *K_d_ =* | *[CD]* | *D_b_ – D_o_* | *+ [C8-HSL]* | *D_o_ – D_b_* | (2) |
| --- | --- | --- | --- | --- | --- |
|  |  | *D_o_ – D_f_* |  | *D_b_ – D_f_* |  |

Since diffusion coefficients are sensitive to variations of temperature and sample viscosity, comparisons across samples are difficult. However, the observed diffusion coefficients (*D_o_*) can be corrected using the diffusion of water in each sample and comparing that to a standard value. For this study we corrected each *D_o_* with that of the H_2_O measured in the solution of only C8-HSL.

*D_Corr_ = D_o_ D_H2O_ / D_H2O-C8-HSL_* (3)

The calculated values of K_d_ for each of the solution are included in Supplementary Table 1. The three values for the β‑CD are in close agreement with each other (average = 1.50 x 10^-3^ M). This value is 7.5 times higher than that of the α‑CD. Expressed differently, C8-HSL binds 7.5 times stronger in the α‑CD than the β‑CD (*K_a_* = 1/*K_d_*). The smaller interior of the α‑CD is a better fit for the C8-HSL. This procedure was also used to determine the diffusion coefficient for 3OC6-HSL and β-CD; however, initial attempts were unsuccessful and did not indicate binding.

**Supplementary Table 1** Measured and corrected diffusion coefficients (*D*) for various mixtures of C8-HSL and cyclodextrin. The dissociation constant (*K_d_*) is calculated from equation 2. All solutions were prepared in D_2_O.

| **Solution composition** | **Measured *D* x10^-10^ m^2^s^-1^** | | | **Corrected  *D* x10^-10^ m^2^s^-1^** | | ***K_d_ 10^-3^ M*** |
| --- | --- | --- | --- | --- | --- | --- |
|  | **C8-HSL** | **α/β-CD** | **H_2_O** | **C8-HSL** | **α/β-CD** |  |
|  |  |  |  |  |  |  |
| 10 mM C8-HSL | 3.55 |  | 13.33 |  |  |  |
|  |  |  |  |  |  |  |
|  |  |  |  |  |  |  |
| 5.3 mM C8-HSL + 23 mM α-CD | 2.00 | 2.00 | 13.62 | 1.96 | 1.96 |  |
| 8 mM C8-HSL + 8 mM α-CD | 2.24 | 2.04 | 12.68 | 2.35 | 2.14 | 0.21 |
|  |  |  |  |  |  |  |
|  |  |  |  |  |  |  |
| 2 mM C8-HSL + 2 mM β-CD | 2.79 | 1.98 | 12.79 | 2.91 | 2.06 | 1.49 |
| 3 mM C8-HSL + 2 mM β-CD | 2.88 | 1.92 | 12.89 | 2.98 | 1.99 | 1.57 |
| 4 mM C8-HSL + 2 mM β-CD | 2.97 | 1.93 | 13.04 | 3.04 | 1.97 | 1.44 |

**Supplementary Table 2** Binding strength of β-CD and C6-HSL or C8-HSL as determined by NMR.

|  | **C6-HSL** | **C8-HSL** |
| --- | --- | --- |
| **Binding constant** | 0.11 | 0.69 |
| **Dissociation constant** | 9.05 | 1.44 |
| **Percent bound** | 17 | 35 |


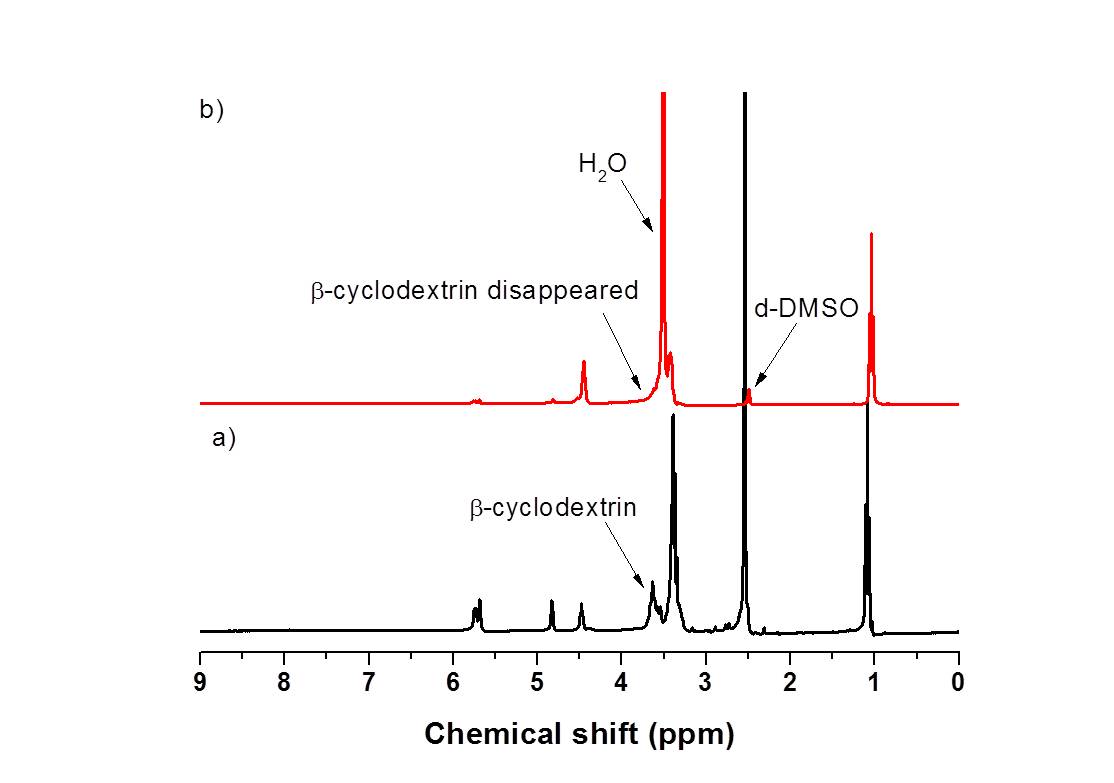


**Supplementary Figure 1** ^1^H NMR spectra of the as-synthesized β-CD coated silica nanoparticles. a) before dialysis; b) after dialysis.





**Supplementary Figure 2** TGA of (a) dye-labeled monolayer carboxylic acid coated silica nanoparticles; (b) dye-labeled monolayer β-CD coated silica nanoparticles. Graft density was 0.27 groups/nm^2^ for 15 nm Si-NPs and 0.11 groups/nm^2^ for 50 nm Si-NPs.


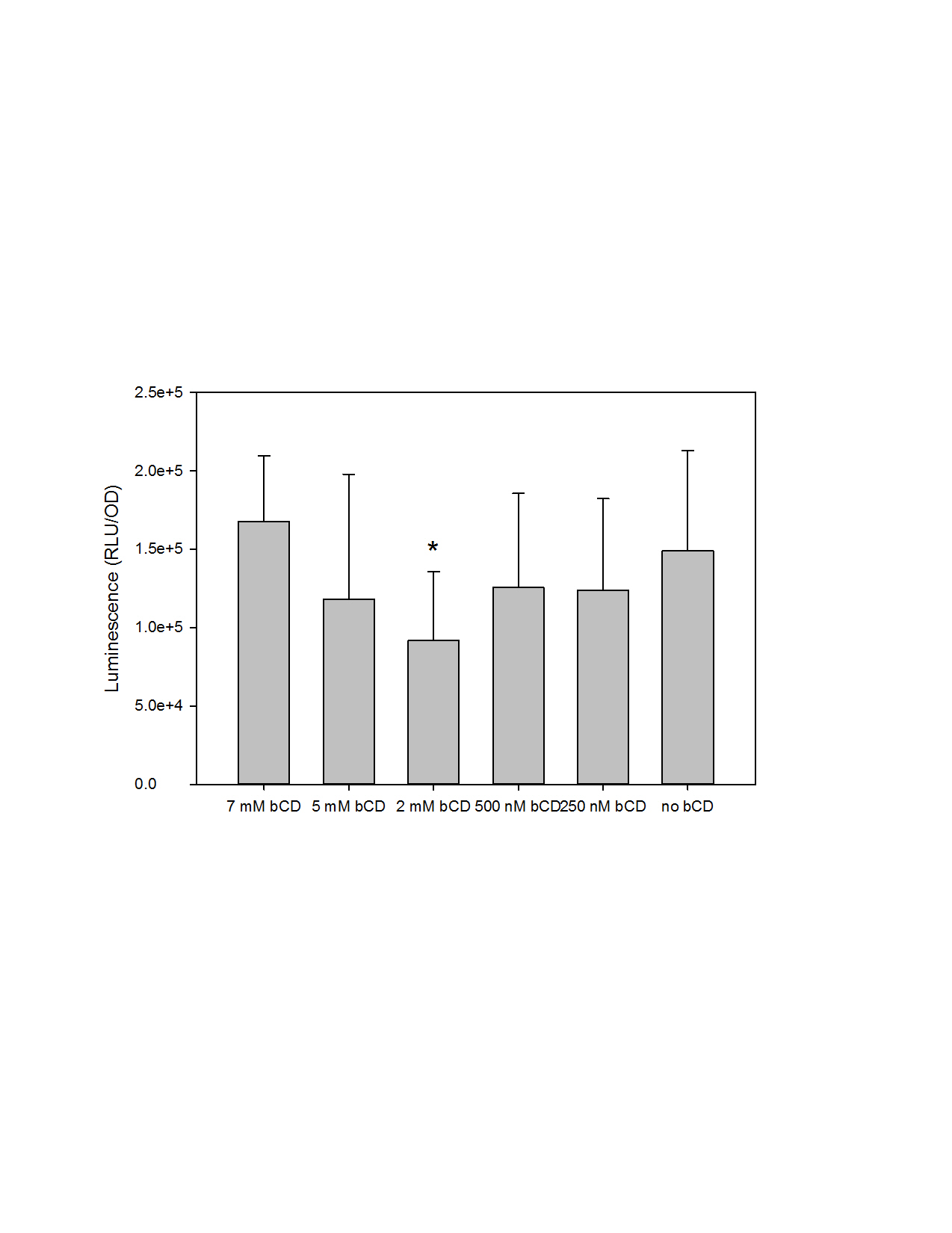


**Supplementary Figure 3** Maximum relative bioluminescence per OD 600 nm of *V. fischeri* during exposure to β-CD and 2 μM 3OC6-HSL. Error bars represent standard error of the mean. Asterisk indicates significance (P≤ 0.05).
